# Geroprotection through modulation of heart rate variability, oxidative enzymes, tissue integrity, and gene expression by an Ayurvedic herbal formulation, Amalaki Rasayana

**DOI:** 10.1016/j.jaim.2026.101338

**Published:** 2026-05-22

**Authors:** Cheryl Rhea Lewis, Vasudha Devi, Dinesh Upadhya, Dhandapani Kuppuswamy, Rajshekhar Chinta, Shiny Jasphin, Souvik Dey, Neha Choudhari, Anandan E M

**Affiliations:** a Department of Pharmacology, Kasturba Medical College, Manipal, Manipal Academy of Higher Education, Manipal, 576104, Karnataka, India; b Division of Pharmacology, Department of Basic Medical Sciences, Manipal Academy of Higher Education, Manipal, 576104, Karnataka, India; c Centre for Molecular Neurosciences, Kasturba Medical College, Manipal, Manipal Academy of Higher Education, Manipal, 576104, Karnataka, India; d Department of Medicine, Medical University of South Carolina, Charleston, SC, 29425, USA; e Department of Pharmacology, Manipal University College Malaysia, Melaka, 75150, Malaysia; f Aaray Health Solutions Private Limited, GoK Bio-incubator, Manipal Academy of Higher Education, Manipal, 576104, Karnataka, India; g Department of Biotherapeutics Research, Manipal Academy of Higher Education, Manipal, 576104, Karnataka, India; h Arya Vaidya Sala, Kottakkal, 676503, Kerala, India; i Centre for Cardiovascular Pharmacology, Manipal Academy of Higher Education, Manipal, Karnataka, India

**Keywords:** Geroprotection, Anti-aging, Amalaki rasayana, Ayurveda, Traditional medicine

## Abstract

**Background:**

Aging is a multifactorial process involving cumulative cellular and organ deterioration, largely due to oxidative stress and disrupted homeostasis. These changes lead to increased susceptibility to age-related diseases. Amalaki Rasayana (AR), an ayurvedic formulation from the fruit of *Phyllanthus emblica*, is traditionally valued for its geroprotective, rejuvenating effects and preventive healthcare. **Objective:** This study scientifically evaluates AR's impact on physiological, biochemical, and molecular markers of aging and functional decline in a rodent model.

**Methods:**

Thirty-six, 10 months old, male Fischer rats, (n = 6) were randomized into normal control (NC) and Amalaki Rasayana (AR) -treated groups, and evaluated at the end of 18, 24, and 30 months of age. Comprehensive assessments including, electrocardiography, histopathology, biochemical assays, and molecular analyses were conducted to evaluate cardiac, renal, hepatic, and neural tissue health. Oxidative stress was quantified by measuring superoxide dismutase (SOD) and catalase activity. Tp53 and p21 gene expressions were quantified by qPCR and bioinformatics.

**Results:**

Our results demonstrated pronounced age-associated degenerative changes such as neuronal loss, myocardial fibrosis, renal tubule dilation and glomerular fragmentation in NC rats. In contrast, AR-supplementation maintained heart rate variability, preserved antioxidants superoxide dismutase and catalase activities, and mitigated tissue degeneration across multiple organs. AR modulated the expression of Tp53 and p21 in cardiac and neural tissues, suggesting a role in cellular stress response and longevity pathways.

**Conclusion:**

Amalaki Rasayana demonstrated potent antioxidant and cytoprotective effects in aging rats, supporting its potential as an adjuvant for healthy aging. These findings highlight AR's promise in attenuating oxidative damage and modulating gene expression, warranting further translational research.

## 1 Introduction

The growing impact of age-related diseases has intensified research into strategies for healthier aging. Ayurveda, India's ancient medical system, has addressed healthy aging for thousands of years. As the “science of longevity,” Ayurveda outlines strategies in foundational texts such as the Charaka Samhita and Sushruta Samhita, emphasizing harmony with nature, constitution-based therapy, and the prevention of age-related decline [1,2]. While modern medicine has extended life expectancy, the global burden of age-related diseases marked by morbidity, functional decline, and diminished quality of life remains a pressing public health challenge [3,4]. Biological aging, unlike irreversible chronological aging, reflects cellular and molecular deterioration and presents opportunities for intervention [5]. Ayurveda addresses this through rasayanas - specialized rejuvenative formulations designed to slow biological aging and preserve vitality [6]. Among these therapeutic formulations, Amalaki Rasayana (AR), a preparation grounded in the traditional use of *Phyllanthus emblica* (Indian gooseberry), as its principal ingredient, often combined with honey, and ghee, has shown promise against age-related degeneration.

A growing body of preclinical research supports AR's biological relevance, although a significant gap remains in long-term, mechanistic, and translational studies. Studies in Drosophila and rodent models indicate that AR exerts neuroprotective, antioxidative, and longevity effects, enhancing cognitive and physiological resilience [7,8]. Mechanistically, AR has been shown to scavenge free radicals, inhibit collagenase activity, modulate apoptotic and autophagic pathways, and support both cardiac and neuronal function [[9], [10], [11], [12]]. Despite these promising observations, most existing studies are short-term or in vitro, limiting their direct applicability to complex, multifactorial human aging. Long-term AR supplementation has been associated with improved mitochondrial bioenergetics in rodents [11] and telomere maintenance in adults [13], yet evidence addressing dose relevance, midlife initiation, and multiorgan outcomes remains scarce.

To address these gaps, the present study evaluates long-term AR supplementation with endpoints strategically selected to reflect AR's traditional claims of supporting cardiac, neural, and systemic resilience. Heart rate variability (HRV) as a parameter, was measured, as it provides an objective measure of autonomic balance and physiological adaptability induced by AR in response to stress. Measures of tissue fibrosis were included to test the claim that AR preserves structural integrity and delays age-related tissue damage. Oxidative enzyme status was evaluated given AR's long-recognized antioxidant actions, which form the core of its rasayana profile. Tp53 and p21 were quantified as molecular sensors of stress and senescence, enabling direct assessment of AR's purported ability to reduce cellular damage and maintain biological profile.

In Ayurvedic literature, rasayana formulations are traditionally described as promoting systemic rejuvenation and enhancing physiological resilience. Independent of these traditional claims, the present study evaluates the effects of Amalaki Rasayana (AR) using defined biological and molecular endpoints. We hypothesize that long-term, midlife-initiated AR administration attenuates age-related decline in autonomic function and antioxidant enzyme activity through modulation of aging-related gene expression. Initiation of AR at 10 months of age (midlife in rats, approximating 45–50 years in humans) reflects a clinically relevant window, as traditional practice recommends that rasayana therapy begin in midlife, when functional decline typically accelerates and preventive interventions have the greatest translational value.

## 2 Materials and Methods

### 2.1 Animals

**Fig. 1:**
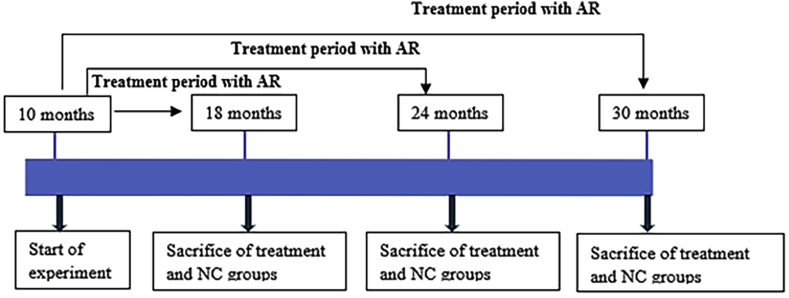
**Study design for anti-aging effect of Amalaki Rasayana in aging rats**. The figure illustrates the schedule of treatment and sample collection across the study duration. Sacrifice and tissue collection from both treatment and normal control (NC) groups were conducted at the end of 18, 24, and 30 months, enabling the assessment of organs at distinct time points. This design allowed for comparative evaluation of changes over time between groups.

Thirty-six male Fischer-344 rats, aged 10 months, were obtained from the Central Animal Research Facility of Kasturba Medical College (KMC), Manipal and were randomly allocated to either the treatment or control group, with cohorts designated for follow-up at the end of 18, 24, and 30 months of age. Each group comprised n = 6 animals, reflecting established guidelines for aging studies and following a protocol approved by the Institutional Animal Ethics Committee (Ref: no. IAEC/KMC/72/2019). Amalaki Rasayana (AR) was administered orally to animals of the treatment groups for five days a week at a dose of 500 mg/kg body weight as a 0.4 mL oral solution (Fig. 1, Table 1). Every animal was kept in a typical laboratory setting with a 12-h light/dark cycle, 22–24 °C, and unlimited access to food and water. At the end of the designated experimental periods, animals from each group were euthanized under deep thiopentone anesthesia. Post-mortem, organs including the heart, kidneys, liver, and brain were excised, fixed in formalin, and subsequently processed for histopathological analysis.

The study commenced when animals reached 10 months of age, at which time AR treatment was initiated and maintained for the AR groups. Sequential assessments were conducted at 18, 24, and 30 months, following which animals from both the treatment and normal control (NC) groups were sacrificed at the end of their pre-determined timepoints. These predefined time points enabled evaluation of early, intermediate, and late senescent changes as well as the effects associated with AR treatment as age advanced. At each interval, comprehensive physiological, biochemical, and tissue analyses were performed to characterize treatment-related changes over the course of the study.

*Dosage:* The rationale for the selected AR dose in this study matches previously validated animal studies where doses in the 500–750 mg/kg range have shown comparable physiological benefits, with 500 mg/kg identified as both effective and safe, especially in long-term studies. This dose was found to produce significant biological effects, including improved endurance, mitochondrial bioenergetics, and antioxidant protection without causing adverse reactions [11]. The regimen of 5 days per week reflects chronic, yet minimally disruptive administration [14], mimicking traditional cyclic use and allowing for recovery periods as per Ayurvedic guidelines. This dosing protocol thus provides a balance between efficacy, tradition, and safety for evaluating long-term effects in aging models.

### 2.2 Experimental formulation

Arya Vaidya Sala, a GMP-certified facility in Kottakkal, Kerala, India, prepared and provided Amalaki Rasayana (AR). The Centre for Medicinal Plants Research and the internal Quality Assurance department confirmed the authenticity of the Indian gooseberry, *Phyllanthus emblica,* that was used. Dried fruit powder and fresh juice from the fruit were dried (55 °C, 700 mmHg) and combined for 20 cycles in accordance with the preparation process described in Ayurvedic texts (Charaka Samhita) and by Kumar et al. [11]. Bioavailability was improved by a final (21st) trituration. In order to ensure stability, nutritional value, and therapeutic efficacy, the product was blended in a 1:2:0.5 ratio with honey and clarified butter (ghee).

### 2.3 Electrocardiograph (ECG) recording

The iWORX BIO4 instrument was used to record ECG in anesthetised rats. Subcutaneous needle electrodes were placed in the limbs of rats to enable ECG recording for a duration of 180s from lead I. The resulting waveforms and individual ECG parameters were analyzed using LabScribe software 2.0 for precise interpretation.

### 2.4 Heart rate variability (HRV) analysis using Kubios software

RR intervals were extracted for spectral analysis and categorized into three frequency bands. Because rodents exhibit substantially higher resting heart rates and more rapid autonomic oscillations than humans, their HRV frequency bands are shifted to higher ranges than conventional human standards. Accordingly, the frequency ranges were defined as: very low frequency (VLF: <0.15 Hz), low frequency (LF: 0.15–0.6 Hz), and high frequency (HF: 0.6–2.5 Hz). In this physiological context, the HF band reflects predominantly vagal (parasympathetic) activity, whereas the LF band reflects combined sympathetic and parasympathetic modulation, providing a rodent-appropriate index of autonomic balance.

### 2.5 Histopathology of tissues

Hematoxylin and Eosin (H&E) staining was used to evaluate histopathological alterations in the brain, liver, kidney, and heart. Formalin-fixed tissues were processed, embedded in paraffin, and cut into 5-μm sections for mounting and staining. Similarly, sections of the brain and heart were stained with Masson's Trichrome to assess fibrosis and collagen deposition, while Nissl granules and neuronal cell bodies were visualized using Cresyl Violet staining. To ensure objectivity and strengthen the reliability of interpretations, all histological examinations and qualitative scoring were performed by two independent pathologists blinded to the treatment groups. Sections were examined using an Olympus BX50 microscope at 10X and 40X magnifications, and ImageJ analysis was utilized by blinded researchers to quantify structural changes across treatment cohorts.

### 2.6 Biochemical analysis of SOD and catalase

Lysates from heart and brain tissues were analyzed to assess the activity of key antioxidant enzymes - superoxide dismutase (SOD) and catalase using assay kits from Sigma Aldrich (product nos: 19160, CAT100).

### 2.7 qPCR for expression levels of senescent genes Tp53 and p21

For qPCR analysis, RNA was extracted from heart and brain tissues using an appropriate RNA isolation kit. Complementary DNA (cDNA) was synthesized from 1 μg of RNA using the Prime Script RT Reagent Kit from TaKaRa. Quantitative PCR (qPCR) was performed using gene-specific primers for Tp53 and p21 along with a housekeeping gene (18s) as an internal control. The reaction was carried out in a real-time PCR system using SYBR Green chemistry. Relative gene expression was calculated using the 2^-ΔΔCt method, normalizing target gene expression to the housekeeping gene. The expressions were compared across age groups in normal control and treatment groups.

## 3 Results

### 3.1 Heart rate Variability parameters and sympathetic-parasympathetic modulation (please refer to supplementary file 4)

**Fig. 2:**
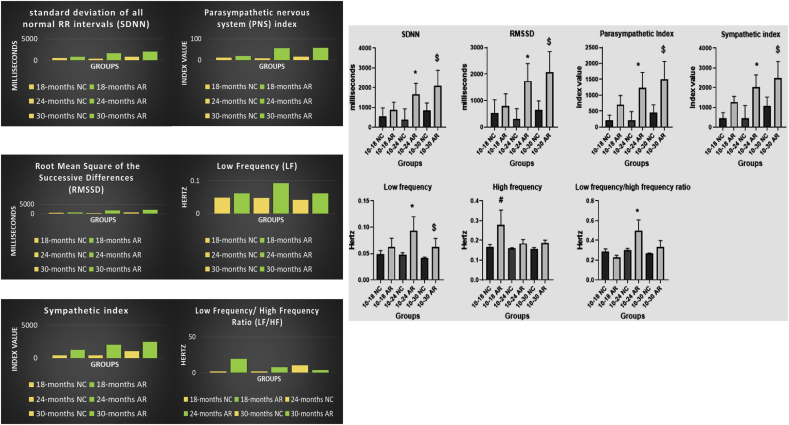
**Graphical representation of effect of Amalaki Rasayana on heart rate variability (HRV) parameters**. The time – dependent graphs show enhanced autonomic balance with significant effects on, standard deviation of all normal-to-normal intervals (SDNN) and root mean square of the successive differences (RMSSD), sympathetic, parasympathetic modulation. p significance legend (p ≤ 0.05): # - 10-18 NC vs 10-18 AR, ∗ - 10-24 NC vs 10-24 AR, $ - 10-30 NC vs 10-30 AR

The 10–30-month AR group demonstrated higher RMSSD and SDNN values compared to age-matched controls, along with an elevated parasympathetic index. The 10–18 NC group exhibited a higher LF/HF ratio relative to the corresponding AR-treated group. (Fig. 2).

### 3.2 ECG parameters and aging (please refer to supplementary file 4)

**Fig. 3:**
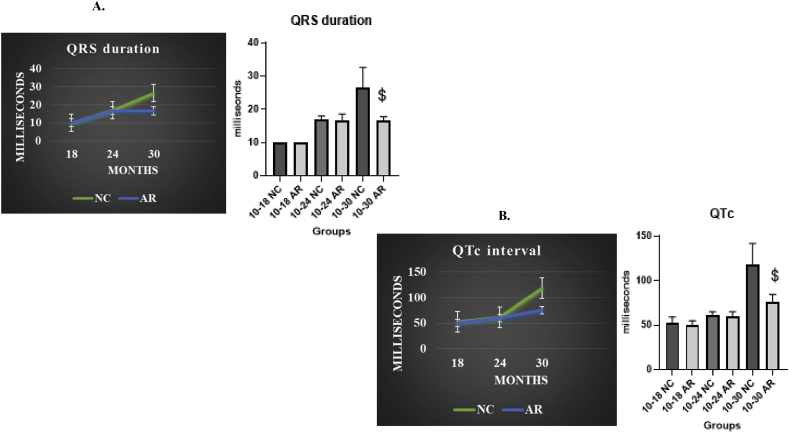
**Effect of Amalaki Rasayana on ECG**. Amalaki Rasayana treatment in aging rats significantly improved QRS duration (P = 0.0074) (3A) and QTc interval (P = 0.0051) (3B) compared to controls, demonstrating enhanced cardiac function and potential cardioprotective effects during aging. Fig. 3A depicts QRS duration, and Fig. 3B QTc interval for NC (green) and AR-treated (blue) groups. The bar graphs present mean values ± standard deviation. Notably, AR-treated animals demonstrated significant improvements in QRS duration and QTc interval (p < 0.05) compared to the NC group. (For interpretation of the references to colour in this figure legend, the reader is referred to the Web version of this article.)

Both groups experienced a minor prolongation of QRS duration (Fig. 3A), consistent with age-related structural changes in the ventricular conduction system. The control group showed a significant age-related increase in QTc intervals, whereas this increase was attenuated in the Amalaki Rasayana (AR)-treated group. (Fig. 3B).

### 3.3 Staining and Magnification of Hippocampal subfields at the end of 18 months (please refer to supplementary file 4)

**Fig. 4:**
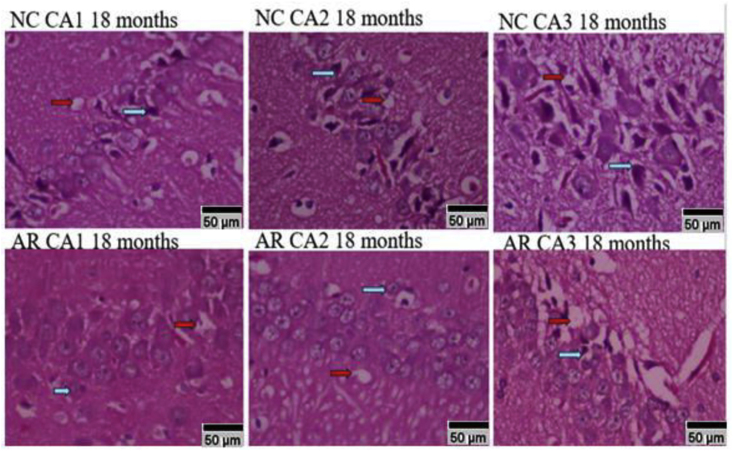
**Staining and 10× Magnification of Hippocampal subfields at the end of 18 months.** The sections reveal that the neuronal architecture in the AR group remained largely preserved, showing close similarity to the normal control (NC) group. Degenerating or lytic neurons are marked with red arrows, while intact, morphologically normal neurons are marked with blue arrows, allowing a clear visual comparison of cellular integrity across groups. (NC: Normal control; Amalaki Rasayana: AR). (For interpretation of the references to colour in this figure legend, the reader is referred to the Web version of this article.)

Comparison between the two groups at the end of 18 months, revealed that the NC group exhibited vacuolation (Fig. 4, red arrow) and neuronal lysis (Fig. 4, blue arrow), particularly within the CA2 and CA3 regions, with CA3 being the most affected.

### 3.4 Cresyl violet staining of Hippocampal subfields and dentate gyrus at the end of 24 and 30 months at 10× and 40× magnifications (please refer to supplementary file 4)

**Fig. 6:**
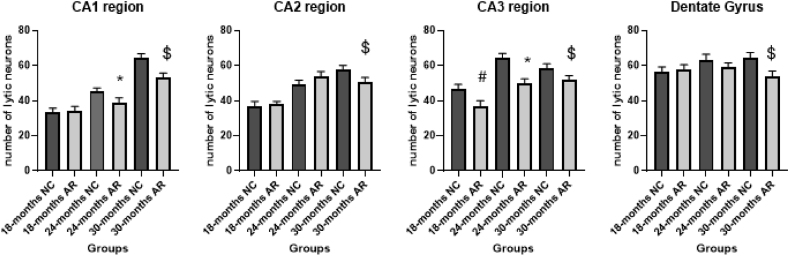
**Statistical correlations of lytic neurons in hippocampal subfields from 18 to 30 months in NC and AR groups**. Statistical significance for CA1 subfield: ∗P - 0.0047 (p ≤ 0.01) 10-24 NC vs 10-24 AR, $ P – 0.001 (p ≤ 0.001) 10-30 NC vs 10-30 AR. CA2 subfield: $ P – 0.0017 (p ≤ 0.001) 10-30 NC vs 10-30 AR. CA3 subfield: #P - 0.001 (p ≤ 0.001) 10-18 NC vs 10-18 AR, ∗P - 0.001 (p ≤ 0.001) 10-24 NC vs 10-24 AR, $ P - 0.008 (p ≤ 0.01) 10-30 NC vs 10-30 AR. CA4 subfield: $ P – 0.001 (p ≤ 0.001) 10-30 NC vs 10-30 AR.

The normal control (NC) group animals showed a higher density of darkly stained, lytic neurons (Figs. 5 and 6). AR-treated animals displayed a higher number of healthy neurons across all the time points. Neural integrity scores declined from 85% at 18 months to 40% by 30 months for the NC group while integrity scores, declined from 95% at 18 months to 88% at 30 months in the AR treated group (supplementary file 2).

### 3.5 Fibrosis in the aging myocardium from 18 - 30 months and percentage increase in myocardial fibrosis in left ventricle from 10-30 months (please refer to supplementary file 4)

**Fig. 7:**
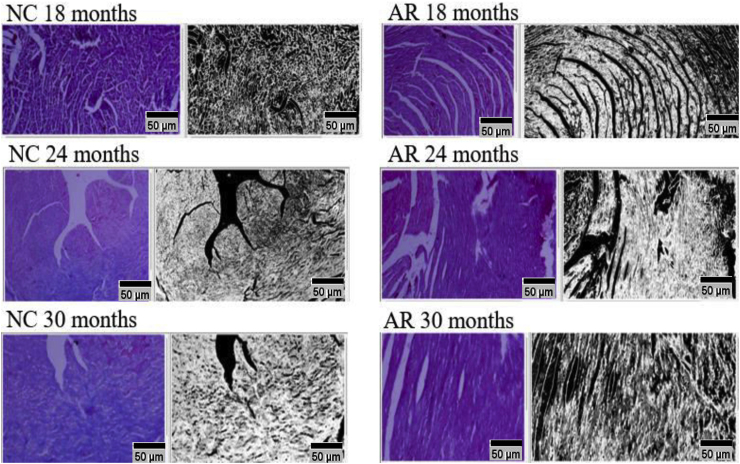
**Fibrosis in the aging myocardium from 18**–**30 months**. Histological analysis of myocardial tissue fibrosis in normal control (NC) and Amalaki Rasayana (AR)-treated groups at 18, 24, and 30 months. Representative sections of myocardial tissue stained with Masson's trichrome and corresponding grayscale processed images demonstrate fibrosis (fibrotic areas appear as white following ImageJ analysis) and its progression in NC and AR groups from 18 months to 30 months.

**Fig. 8:**
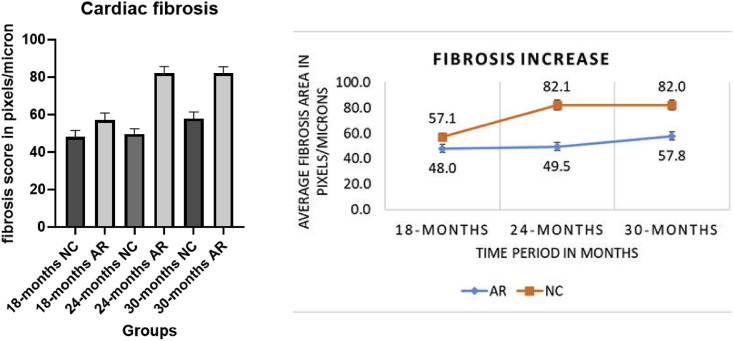
**Represent statistical significance and percentage increase in myocardial fibrosis from 10**–**30 months**. #P - 0.01 (p ≤ 0.01) 10-18 NC vs 10-18 AR, ∗P - 0.001 (p ≤ 0.001) 10-24 NC vs 10-24 AR, $ P - 0.001 (p ≤ 0.01) 10-30 NC vs 10-30 AR

Diffuse fibrosis was present in both groups starting at 18 months, the NC group exhibited a sharp, widespread increase that reached statistical significance by 30 months. The AR-treated group showed an attenuated, gradual progression, with a lower percentage of fibrotic accumulation compared to the control group (Fig. 7, Fig. 8).

### 3.6 H&E staining and changes in glomerular architecture of kidneys in NC and AR-treated groups (please refer to supplementary file 4)

**Fig. 9:**
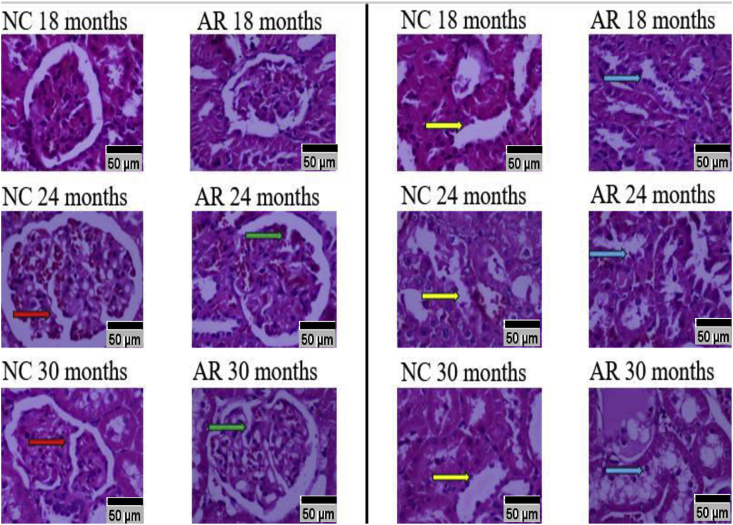
**Aging changes in glomerulus and convoluted tubules of kidneys at 40× magnification.** Comparison of renal histology (18–30 months) shows progressive Bowman's space expansion and glomerular fragmentation (red, green arrows). Proximal tubules reveal lumen dilation in NC (yellow arrows), whereas AR-treated rats exhibit age-related tubular enlargement (blue arrows). (For interpretation of the references to colour in this figure legend, the reader is referred to the Web version of this article.)

**Fig. 10:**
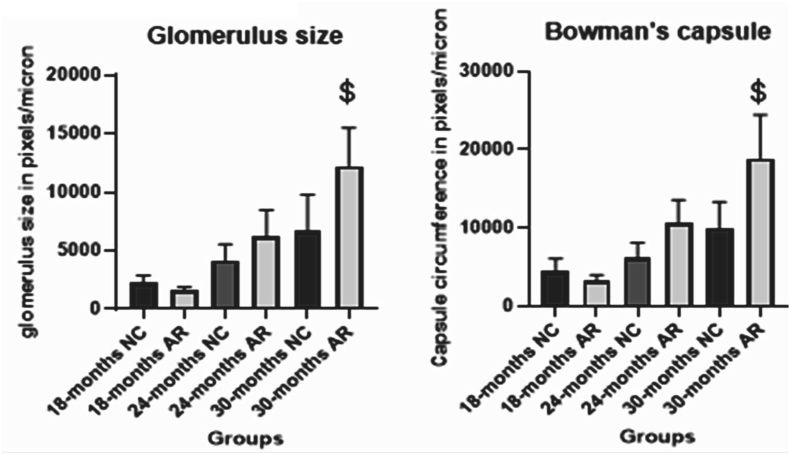
**Comparative analysis of glomerular architecture in NC and AR-treated groups.** Glomerulus size: $ P = 0.009 (30-months NC vs 30-months AR); Bowman's capsule: $ P value = 0.0015 (30-months NC vs 30-months AR). (NC: Normal control; Amalaki Rasayana: AR).

At 30 months, both the NC and AR-treated groups exhibited tubular enlargement and widened interstitial spaces (Fig. 9). The NC group showed greater tubular enlargement and widened interstitial spaces compared to the AR-treated group. (Fig. 10).

### 3.7 Oxidative enzymes in heart and brain tissues in NC and AR-treated animals

**Fig. 11:**
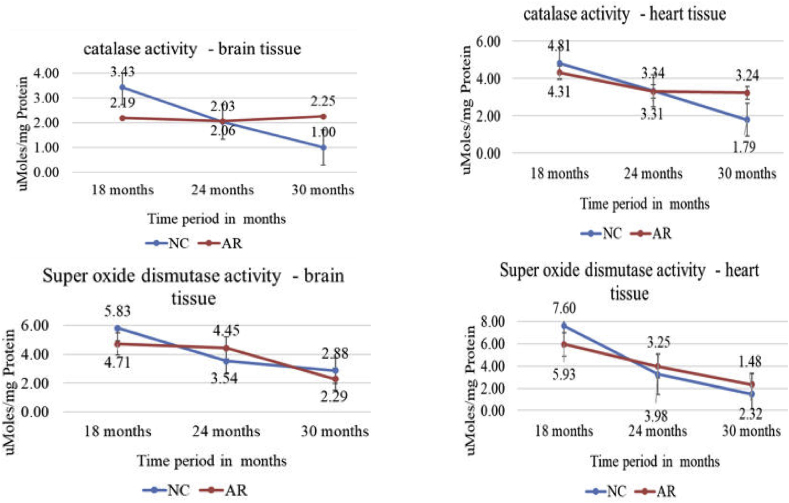
**Trendline of oxidative enzymes in heart and brain tissues.**Fig. 11 shows catalase and SOD activity in the brain and heart tissues. Both declined with age, but NC rats (represented by the blue line) exhibited greater loss in antioxidant activity, while AR treatment (represented by the red line) mitigated decline, particularly in the heart tissue. (For interpretation of the references to colour in this figure legend, the reader is referred to the Web version of this article.)

Oxidative enzyme analysis revealed an age-dependent decline in SOD and catalase activities across both heart and brain tissues (Fig. 11). In cardiac tissue, NC group values were lower than AR-treated group values after 24 months, while in brain tissue, NC group values were lower than AR-treated group values at all time points. This pattern was observed across both the tissues.

### 3.8 Molecular Interactions of Tp53 and p21(Cdkn1a) – genes involved in aging and functional enrichment Analysis through STRING network (please refer to supplementary file 4)

**Fig. 12:**
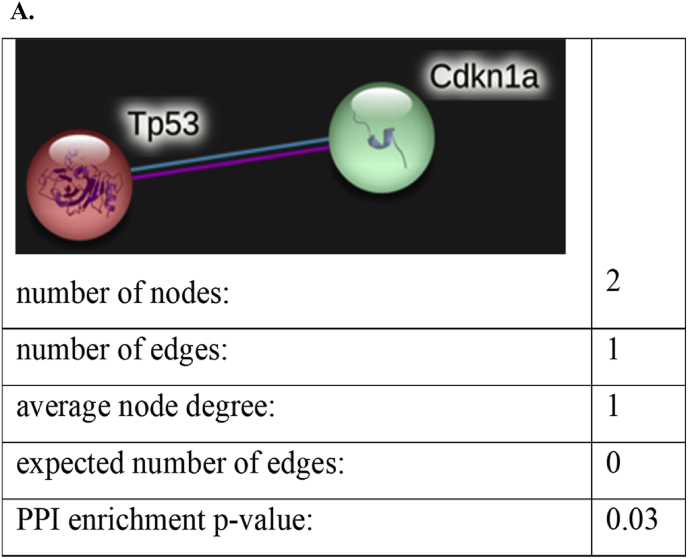
A. Molecular Interactions of p53 and p21 – genes involved **in aging.** Cytoscape-based STRING network analysis we mapped the molecular interactions involving Tp53 and p21. The analysis highlights robust associations between Tp53 and cyclin-dependent kinase inhibitors, such as p21 which propagate senescence.

The STRING network analysis of interaction between Tp53 and p21 was statistically significant (PPI enrichment p = 0.03) (Fig. 12A), indicating biological relevance rather than a coincidental correlation. Their coordinated response to cellular stress and DNA damage, which is essential for aging and tumor suppression, is supported by the direct Tp53–p21 connection. The strength of their involvement was further validated by STRING-based functional enrichment analysis (), confirming their pivotal role in molecular mechanisms associated with aging.

**Table 1:** Illustrates the schedule of treatment and sample collection across the study duration.

Group (n = 6)	Duration of experiment		Drug Dose and route
	Age of animals at the start of experiment (months)	Age of animals at the end of experiment (months)	
1	10 months	18 months	AR 1 g/kg/day (0.4 ml solution) for 5 days/week orally.
2	10 months	24 months	
3	10 months	30 months	

### 3.9 Tp53 and p21 expressions in brain and heart tissue (please refer to supplementary file 4)

**Fig. 13:**
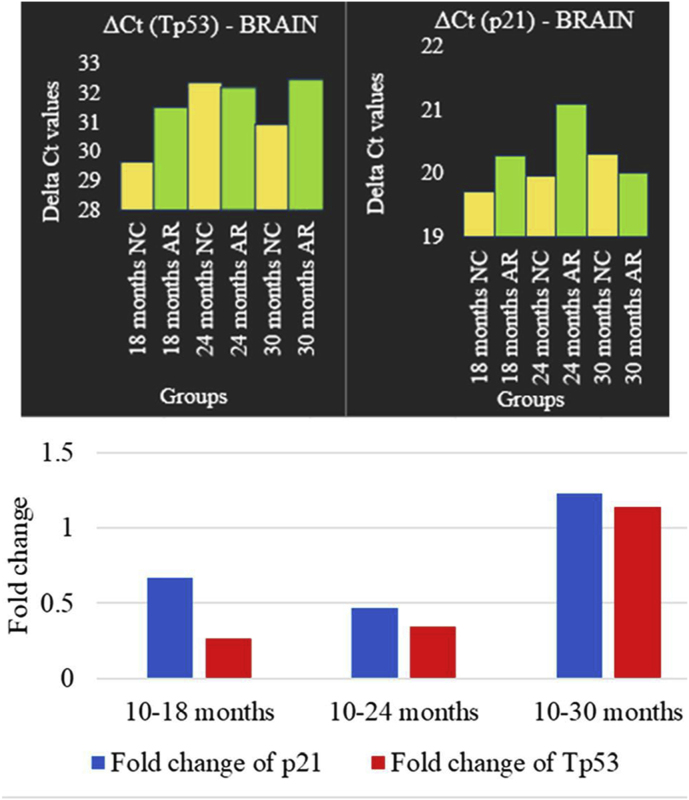
**Groupwise comparison of Tp53 and p21 gene expression in brain tissue.** Bar plots display the ΔCt values and fold changes of Tp53 and p21 gene expression in brain tissue for control and AR-treated groups at 18, 24, and 30 months. Comparative trends demonstrate age-dependent variation in both Tp53 and p21 expression following AR treatment. p21 (blue) shows an increased expression from younger to older animals. Tp53 (red) is notably suppressed in younger (18 months) and middle age (24 months) groups, with an increase in older age (30 months). (For interpretation of the references to colour in this figure legend, the reader is referred to the Web version of this article.)

Neural expression - Quantitative PCR (qPCR) was utilized to evaluate the expression of senescence markers Tp53 and p21 in brain tissue at the end of 18, 24, and 30 months (Fig. 13). At 18 and 24 months, ΔCt values were higher in the AR-treated group than in the NC group. Fold-change analysis showed Tp53 expression values of ≈0.27 at 18 months, ≈0.34 at 24 months, and ≈1.14 at 30 months. p21 expression followed a comparable pattern.

**Fig. 14:**
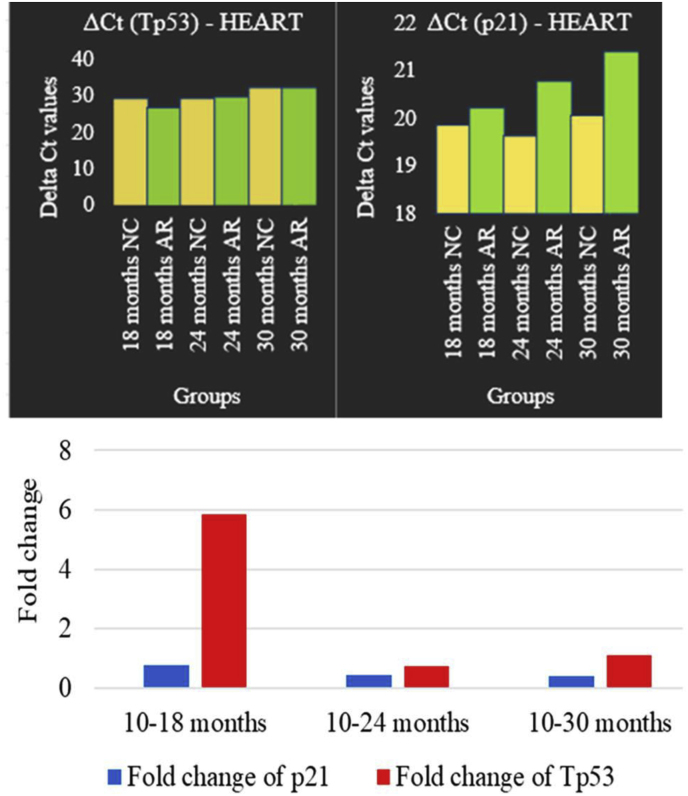
**Groupwise comparison of Tp53 and p21 gene expression in heart tissue.** Graphs illustrate the ΔCt values and fold changes of Tp53 and p21 gene expression in heart tissue across control and AR-treated groups at 18, 24, and 30 months. The data highlight myocardium-specific changes in Tp53 and p21 expression, revealing distinct regulation profiles in response to AR treatment over time. p21 (blue) consistently decreases with age while Tp53 (red) shows an increase at 18 months, then declines at middle age with a slight increase towards old age (30 months). (For interpretation of the references to colour in this figure legend, the reader is referred to the Web version of this article.)

Cardiac expression - Quantitative PCR analysis of heart tissue revealed that AR treatment increased ΔCt values compared to the NC group (Fig. 14 ). Tp53 showed early suppression at 18 months (fold change ≈ 0.18), moderate at 24 months (≈0.32) and 30 months (≈0.56). p21 expression values were ≈0.54, ≈0.40, and ≈0.33 at 18, 24, and 30 months, respectively.

## 4 Discussion

Aging is characterized by numerous physiological changes that impact various organ systems, including the autonomic nervous system (ANS), cardiovascular health, liver, renal function, and neuronal integrity. In this study, we investigated the effects of Amalaki Rasayana (AR) on age-related changes in these systems, providing valuable insights into its prophylactic potential for mitigating the adverse effects of aging.

### 4.1 Autonomic regulation, heart rate variability, and the heart-brain axis in aging

The heart–brain axis operates through tightly coordinated neural, hormonal, and autonomic pathways, with HRV serving as a sensitive marker of systemic adaptability [15]. As a regulator of sympathetic–parasympathetic balance, the ANS plays a central role in maintaining cardiovascular stability and stress resilience across aging [16]. Within this regulatory context, the study revealed distinct autonomic patterns across groups. The 10–30 months AR cohort exhibited higher SDNN in later months, consistent with enhanced stress-resilience capacity [17], while LF power peaked in the 10–18 months AR group. HF power, indicative of vagal modulation, was greatest in the 10–18 months NC and 10–30 months AR groups. An elevated LF/HF ratio in the 10–18 months AR group aligned with AR's known inotropic actions [11]. Overall, these autonomic signatures support the interpretation that AR promotes midlife autonomic balance and resilience, aligning with its reported adaptogenic properties [18].

### 4.2 ECG and cardiac histology

Age-related changes due to cardiomyocyte loss, hypertrophy, and fibrosis, manifest as prolonged QTc intervals and P wave durations in ECG, highlighting increased cardiac vulnerability [[19], [20], [21]]. Graphs of QRS duration and QTc intervals in our study showed NC rats had progressive QRS prolongation and greater QTc increases as age advanced, while AR-treated rats maintained stable conduction parameters. Histological analysis correlated this to increased myocardial fibrosis in NC rats, especially after 24 months, whereas AR-treated animals exhibited slower progression of fibrosis and less fibrotic tissue. Collectively, these findings support a cardioprotective effect of AR, suggesting its potential to prevent age-related fibrosis and preserve myocardial function [11].

### 4.3 Aging and Neuroprotection of AR (supplementary file 2)

Age-related hippocampal decline is driven by cumulative metabolic stress, impaired neurotrophic signalling, and reductions in synaptic plasticity, which render regions such as CA2 and CA3 particularly susceptible to degeneration [22,23]. These vulnerabilities provide a mechanistic basis for the progressive deterioration observed in untreated aging rats. In contrast, the comparatively preserved neuronal architecture in the AR-treated groups in our study aligns with evidence that AR can enhance cellular resilience by stabilizing neuronal membranes, reducing cytoplasmic stress, and supporting synaptic maintenance [24]. Experimental studies further suggest that AR modulates glutamatergic and GABAergic pathways and promotes dendritic spine integrity, offering a plausible biological explanation for the attenuated neurodegenerative changes observed in treated animals without reiterating the specific morphological details reported earlier [12,25].

### 4.4 Renal histology and oxidative enzymes

Although the renal medulla generally remains preserved with age, aging kidneys commonly show tubular degeneration, interstitial fibrosis, and glomerular sclerosis [26]. By 24 months, both AR and NC-treated rats exhibited wider Bowman's space, glomerular fragmentation, and tubular dilatation but to a lesser extent in AR treated. Structural changes in aging tissue are parallelly associated with reduced levels of antioxidant enzymes, such as superoxide dismutase (SOD) and catalase, which contribute to oxidative stress, inflammation, and mitochondrial damage [27,28]. In our study, we found that at 24 months, cardiac tissue showed a greater decline in these enzymes than brain tissue, particularly in the NC group. Previous work in aged Drosophila demonstrated that AR treatment supports SOD activity and lessens lipid peroxidation [7]. Combined, these findings suggest that AR may help protect against oxidative stress and slow age-related cellular changes.

### 4.5 The roles of Tp53 and p21 in aging: molecular mechanisms and implications

Tp53 and p21 are essential regulators of cellular aging, maintaining genomic stability and dictating how cells respond to stress [29,30]. Tp53, known as the “guardian of the genome,” initiates senescence or apoptosis in damaged cells, while p21 enforces cell-cycle arrest to limit the propagation of those cells [31,32]. Research investigating neuronal and cardiac pathologies have shown that chronic Tp53 activation is connected to neurodegenerative diseases such as Alzheimer's and Parkinson's and persistent p21 upregulation promotes cardiac fibrosis [[33], [34], [35]]. In our study, Amalaki Rasayana (AR) treatment resulted in lower Tp53 and p21 expression in both heart and brain tissues at younger and middle ages, supporting the possibility that AR can blunt age-related stress signalling and delay neurodegenerative and cardiac pathology. These findings align with the Ayurvedic concepts where rasayanas are traditionally believed to promote longevity and resilience. By demonstrating age-dependent modulation of Tp53 and p21 expression, our results create a molecular bridge between classical claims for AR and its observed impact on cellular aging.

### 4.6 Future directions and functional implications

To further validate the prophylactic efficacy of Amalaki Rasayana (AR), future research should transition from structural and molecular observations to comprehensive functional and behavioural endpoints. While our study highlights preserved neuronal architecture, correlating these findings with cognitive assays—such as the Morris Water Maze for spatial memory or Open Field tests for anxiety and locomotion—would provide a holistic view of AR's impact on "lifespan." Furthermore, establishing a dose-response profile across different age cohorts could help optimize therapeutic windows and identify the minimum effective concentration required to elicit its adaptogenic effects. Comparative studies against other established Rasayanas or standard antioxidant therapies (e.g., Vitamin E or Resveratrol) would also be instrumental in defining AR's unique position in the geriatric pharmacological landscape. Finally, longitudinal clinical trials integrating non-invasive biomarkers of the heart-brain axis, such as real-time HRV monitoring in humans, are necessary to translate these promising pre-clinical results into evidence-based anti-aging protocols.

## 5 Limitations of the study

This study has several limitations that should be acknowledged. The sample size, while adequate for detecting physiological and molecular differences, limits the statistical power for more subtle age-related effects. Only male rats were included, preventing evaluation of possible sex-specific responses to AR, which may be relevant given known differences in aging trajectories. Additionally, the absence of behavioural and cognitive assessments restricts interpretation of how the observed structural and molecular protection translates into functional outcomes. Future studies with larger cohorts, inclusion of both sexes, and comprehensive behavioural testing will be essential to strengthen the translational relevance of these findings.

## 6 Conclusion

The overall physiological and molecular profile observed in this study aligns with the geroprotective actions traditionally attributed to rasayana therapy. Amalaki Rasayana (AR) demonstrated these properties by attenuating oxidative stress, reducing myocardial fibrosis, and limiting hippocampal neurodegeneration across the aging trajectory. Its pronounced effects on cardiac tissue suggest a tissue-specific cytoprotective affinity, reflected in preserved structural architecture, suppression of maladaptive remodelling through p21 downregulation, and maintenance of antioxidant enzyme activity. In neural tissue, AR modulated early aging stress signalling in a region- and age-dependent manner, evident through controlled Tp53 and p21 responses, which correspond with classical descriptions of rasayana actions on cognition and mental stability. Collectively, these results position AR as a biologically plausible geroprotective intervention whose effects mirror core Ayurvedic mechanisms of delaying tissue aging, enhancing stress adaptability, and preserving functional capacity across organ systems. Further mechanistic and longitudinal studies are warranted to deepen understanding of these multi-level protective actions.

## Authors' contributions

CRL contributed to the writing of the original draft, methodology, investigation, data acquisition, and analysis. VD was involved in conceptualization of study, study design, interpretation, review, editing and supervision. DU contributed to the study design, interpretation of results and review. DK was responsible for the study design and review. RC and SJ contributed to data acquisition and data interpretation. AEM and SD were involved in data acquisition and interpretation. NC contributed to data acquisition and analysis. The manuscript was reviewed and approved by all authors prior to submission.

## Ethical considerations

The study was approved by the Institutional Animal Ethics Committee of Kasturba Medical College, MAHE, Manipal (Ref: IAEC/KMC/72/2021) and adhered to the ARRIVE guidelines for animal research and reporting of in vivo experiments.

## Declaration of generative AI

The authors confirm that no generative AI tools were used in the writing, analysis, or interpretation of the manuscript.

## Sources of funding

This work was supported by 10.13039/100019305Intramural fund of Manipal Academy of Higher Education, Manipal, Karnataka, India.

## Conflict of interest

The authors declare that they have no known competing financial interests or personal relationships that could have appeared to influence the work reported in this paper.

## Data Availability

The datasets generated and analyzed are available from the corresponding author upon reasonable request.

## Appendix A Supplementary data

Multimedia component 2 Fig. 5. **(supplementary file 3) Representative images of Cresyl violet-stained sections of hippocampal subfields (CA1, CA2, CA3) and the dentate gyrus (DG).** Hippocampal subfields of NC and AR treated animals captured at 10X magnification at the end of 24 months (Fig. 5A), at the end of 30 months (Fig. 5B), and neuronal morphology of dentate gyrus at end of 18 months to end of 30 months (Fig. 5C). Representative 40X magnifications of the same areas (Fig. 5D), 24 months (Fig. 5E) and 30 months (Fig. 5F) revealed that normal control (NC) rats showed a higher prevalence of lytic neurons, as indicated by red arrows, while animals treated with AR had more healthy neurons, identified by blue arrows. (For interpretation of the references to colour in this figure legend, the reader is referred to the Web version of this article.) Multimedia component 2
