# Supplementary material for: Geroprotection through modulation of heart rate variability, oxidative enzymes, tissue integrity, and gene expression by an Ayurvedic herbal formulation, Amalaki Rasayana
Source: J Ayurveda Integr Med. 2026 May 22;17(3):101338. doi: 10.1016/j.jaim.2026.101338 (PMC13217850; doi:10.1016/j.jaim.2026.101338)

**Figure 5 as Supplementary File 3**

**10x magnification – Hippocampal subfields, NC and AR groups, end of 24 months observable difference in structural integrity and neuronal arrangement**

**5A.**


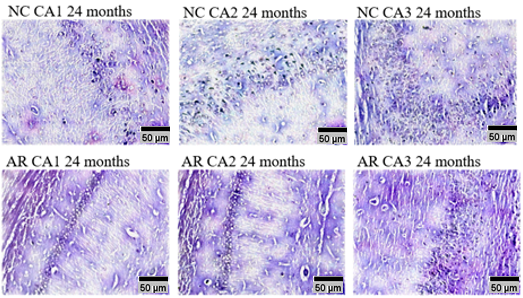


**5B.**

**10x magnification – Hippocampal subfields, NC and AR groups, end of 30 months**


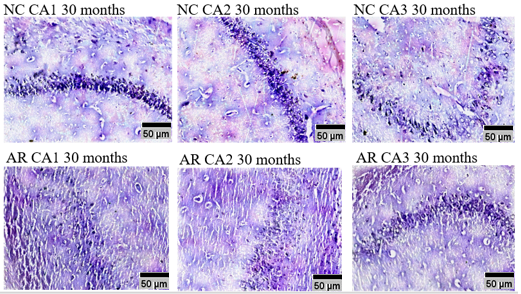


Darkly stained neurons are lytic neurons

**10x magnification – Dentate Gyrus, NC and AR groups, end of 18, 24 and 30months**

**5C.**


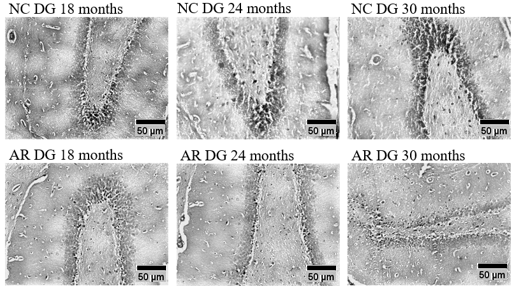


Darkly stained neurons are lytic neurons

**5D.**

**40x magnification – Hippocampal subfields, NC and AR groups, end of 24 months**


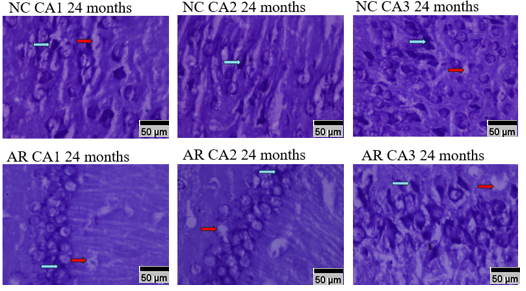


Red arrows - vacuoles; blue arrows – lytic neurons

**5E.**

**40x magnification – Hippocampal subfields, NC and AR groups, end of 30 months**


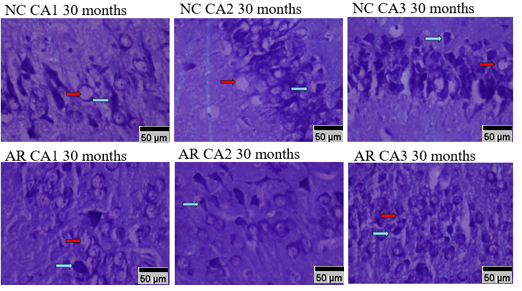


Red arrows - vacuoles; blue arrows – lytic neurons

**5F.**

**40x magnification – Dentate Gyrus, NC and AR groups, end of 18, 24 and 30months**


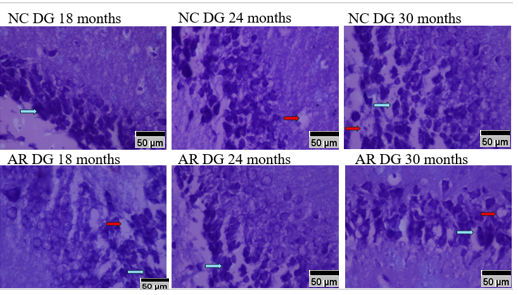


Red arrows - vacuoles; blue arrows – lytic neurons


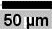

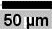

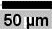

Supplement: Multimedia component 2 — Fig. 5. (supplementary file 3) Representative images of Cresyl violet-stained sections of hippocampal subfields (CA1, CA2, CA3) and the dentate gyrus (DG). Hippocampal subfields of NC and AR treated animals captured at 10X magnification at the end of 24 months (Fig. 5A), at the end of 30 months (Fig. 5B), and neuronal morphology of dentate gyrus at end of 18 months to end of 30 months (Fig. 5C). Representative 40X magnifications of the same areas (Fig. 5D), 24 months (Fig. 5E) and 30 months (Fig. 5F) revealed that normal control (NC) rats showed a higher prevalence of lytic neurons, as indicated by red arrows, while animals treated with AR had more healthy neurons, identified by blue arrows. (For interpretation of the references to colour in this figure legend, the reader is referred to the Web version of this article.) [file mmc2.docx]
