# Supplementary material for: Geroprotection through modulation of heart rate variability, oxidative enzymes, tissue integrity, and gene expression by an Ayurvedic herbal formulation, Amalaki Rasayana
Source: J Ayurveda Integr Med. 2026 May 22;17(3):101338. doi: 10.1016/j.jaim.2026.101338 (PMC13217850; doi:10.1016/j.jaim.2026.101338)
Supplement: Multimedia component 1 [file mmc1.docx]

**3. Results (Supplementary File 4)**

*3.1.* *Heart Rate Variability parameters and sympathetic-parasympathetic modulation*

Heart rate variability (HRV), a measure of the variation in time between two successive heartbeats, showed differences between groups. Two main metrics were examined: standard deviation of normal-to-normal intervals (SDNN), which reflects overall HRV, and root mean square of successive differences (RMSSD), which indicates vagal tone stress state.

*3.2.* *ECG parameters and aging*

QRS duration in an ECG (Figure 3A) measures the time for ventricular depolarization to occur and is influenced by changes in the cardiac conduction system, such as alterations in the Purkinje fiber network and myocardial structure. In the context of this aging experiment, the NC and AR treatment groups showed a slight increase in time taken for electrical impulses to propagate through the ventricular myocardium as age progressed. QTc, which refers to the corrected QT interval, signifies the heart's depolarization and repolarization period and exhibited a steady increase between 10 and 30 months of age in the normal control group, whereas aging animals treated with Amalaki Rasayana showed comparatively shorter QTc intervals over the same period (Figure 3B).

*3.3.* *Staining and Magnification of Hippocampal subfields at the end of 18 months*

In rat models, the 18-month mark represents the transition to early senescence, making H&E staining an essential diagnostic tool for establishing a baseline of structural damage. This stain provides a comprehensive gross overview of hippocampal architecture to determine if the CA1–CA3 layers remain organized or have begun to collapse (Figure 4). Furthermore, H&E is highly effective at identifying acute degenerative markers, such as vacuoles and lytic neurons, which serve as early indicators of active cellular stress and age-related neuronal decay.

*3.4.* *Cresyl violet staining of Hippocampal subfields and dentate gyrus at the end of 24 and 30 months at 10x and 40x magnifications*

Quantitative and qualitative histological assessments of hippocampal subfields demonstrated significant differences in neuronal survival across study groups (Figure 5). High-resolution contrast imaging and automated density analysis via TissueQuant software were employed to quantify neuronal populations, with all counts cross-verified by a blinded pathologist to ensure accuracy. Collectively, these data confirm that Amalaki Rasayana (AR) intervention effectively preserves hippocampal architecture and attenuates age-related neuronal loss.

*3.5.* *Fibrosis in the aging myocardium from 18 - 30 months and* *percentage increase in myocardial fibrosis in left ventricle from 10-30 months*

Masson’s trichrome staining was employed to detect myocardial fibrosis, with qualitative and quantitative analysis performed using TissueQuant software (Figure 7 & Figure 8). The software utilized contrast imaging to isolate fibrotic regions, depicting them as white areas for precise measurement.

*3.6.* *H&E staining and changes in glomerular architecture in NC and AR-treated groups*

Age-related changes in both groups, were characterized by increased inter-tubular spacing, expansion of Bowman’s space, and glomerular fragmentation (Figure 9). These alterations signify a progressive decline in renal structural integrity, where the widening of Bowman’s space indicates glomerular tuft atrophy and increased tubular spacing reflects interstitial fibrosis and tubular loss.

*3.8. Molecular Interactions of Tp53 and p21(Cdkn1a) – genes involved in aging and* *Functional Enrichment Analysis through STRING network* (Figure 12)

| **GO Term ID** | **Term Name** | **p-value** | **Interpretation** |
| --- | --- | --- | --- |
| GO:0051726 | Regulation of cell cycle | 0.00381 | Confirms that the genes regulate the cell cycle. |
| GO:0004861 | Cyclin-dependent kinase inhibitor activity | 0.000000516 | Indicates a strong role in blocking cyclin-dependent kinases |
| GO:0030330 | DNA damage response | 0.000582 | Shows involvement in responding to DNA damage, likely via Tp53-dependent mechanisms. |
| GO:2000647 | Negative regulation of cell cycle process | 0.0146 | Highlights tumor suppressor activity by blocking uncontrolled cell division. |
| GO:0019912 | Cyclin-dependent kinase regulation | 0.0499 | Reinforces that these genes inhibit cell cycle progression. |

**Table 2.** The Gene Ontology enrichment analysis identified terms related to cell cycle regulation, such as 'cyclin-dependent kinase inhibitor activity' and 'DNA damage response'. The extremely low p-values indicate significant enrichment, reinforcing the role of Tp53 and Cdkn1a in controlling cell cycle checkpoints and apoptosis.

*3.9. Tp53 and p21 expressions in brain and heart tissue* (Figure 13 & Figure 14)

| **Age** | **Group** | **p21 expression** | **Fold change (↑↓)** | **Tp53 expression** | **Fold change (↑↓)** |
| --- | --- | --- | --- | --- | --- |
| 10-18 months | AR vs NC | decreased | 0.67↓ | decreased | 0.27↓ |
| 10-24 months | AR vs NC | decreased | 0.47↓ | decreased | 0.34↓ |
| 10-30 months | AR vs NC | increased | 1.23↑ | increased | 1.14↑ |

| **Age** | **Group** | **p21 expression** | **Fold change (↑↓)** | **Tp53 expression** | **Fold change (↑↓)** |
| --- | --- | --- | --- | --- | --- |
| 10-18 months | AR vs NC | decreased | 0.77↓ | increased | 5.85↑ |
| 10-24 months | AR vs NC | decreased | 0.45↓ | decreased | 0.74↓ |
| 10-30 months | AR vs NC | decreased | 0.40↓ | increased | 1.12↑ |

**Table 3.** The dataset above presents and analyses gene expression levels of Tp53 and p21 in the brain over aging periods (10–30 months) with and without AR treatment. The Ct (cycle threshold) values, ΔCt, and ΔΔCt calculations indicate relative gene expression changes.

**Table 4.** The dataset above examines Tp53 and p21 gene expression in heart tissue across aging periods (10–30 months) under AR treatment while the fold-change calculations reflect relative gene expression changes.
